# Marked Reduction of Oxidant Species after Sulfureous Crenotherapy in Females with Joint Diseases and Psoriasis: A Retrospective Real-Life Study

**DOI:** 10.3390/jcm12175731

**Published:** 2023-09-03

**Authors:** Maria Costantino, Valeria Conti, Graziamaria Corbi, Valentina Giudice, Francesco De Caro, Amelia Filippelli

**Affiliations:** 1 Department of Medicine, Surgery and Dentistry “Scuola Medica Salernitana”, University of Salerno, 84081 Baronissi, Italy; vconti@unisa.it (V.C.); vgiudice@unisa.it (V.G.); fdecaro@unisa.it (F.D.C.); afilippelli@unisa.it (A.F.); 2 Association Non-Profit F.I.R.S.Thermae (Interdisciplinary Training, Researches and Spa Sciences), 80070 Bacoli, Italy; 3 Clinical Pharmacology Unit, University Hospital “San Giovanni di Dio e Ruggi d’Aragona”, 84131 Salerno, Italy; 4 Department of Translational Medical Sciences, University of Naples “Federico II”, 80138 Naples, Italy; graziamaria.corbi@unina.it; 5 Italian Society of Gerontology and Geriatrics (SIGG), 50129 Florence, Italy

**Keywords:** free radicals, oxidative stress, crenotherapy, gender, ROMs

## Abstract

Oxidative stress, a condition induced by an excessive amount of free radicals, such as reactive oxygen species (ROS), shows several gender-related differences in basal cellular redox state and antioxidant responses. Crenotherapy with sulfureous mineral water can improve the cellular redox state. In this retrospective observational study, gender-related differences in the efficacy of sulfureous crenotherapy in decreasing oxidant species were investigated. Seventy-eight patients, stratified by sex, with osteoarthritis or degenerative joint disease and Vulgar psoriasis who have received a cycle of sulfureous mud-bath therapy + sulfureous hydropinotherapy were enrolled. Plasma concentration of oxidant species and clinical outcomes were measured at baseline and at the end of treatment. After 2 weeks of sulfureous crenotherapy, a significant amelioration of clinical outcomes and a significant reduction of oxidant species were observed in both sexes, more marked in females than in males (*p* = 0.0001 and *p* = 0.04, respectively). For patients with high oxidant species at baseline, females showed a greater reduction in itching compared to males (−95% vs. −50%), while men had a higher amelioration in pain and morning stiffness (−45% vs. −32%, and −50% vs. −37%, respectively). In conclusion, sulfureous crenotherapy can be a valuable strategy to improve cellular redox state in both sexes.

## 1. Introduction

Oxidative stress is a condition induced by the presence of excessive levels of free radicals, such as Reactive Oxygen Species (ROS), resulting from increased production and/or reduced effectiveness of endogenous antioxidant systems to neutralize them [1,2,3,4]. ROS are physiologically produced by macrophages and leukocytes to kill pathogens; however, an exaggerated production can lead to oxidative damage, tissue dysfunction, and cell death [5]. The principal cytotoxic mechanism of ROS is the induction of detrimental modifications on membrane phospholipids, enzymes, and nuclear proteins, resulting in structural alterations and impaired functions [3,6]. ROS and other free radicals are involved in the pathogenesis of several diseases, such as type 2 diabetes, cardiovascular diseases, cancer, osteoarthritis (OA), and vulgar plaque psoriasis [6].

Gender plays an important role in modulating basal cellular redox state and oxidative stress response, as females are more resistant to oxidative stress, such as chronic oxidative stress induced by iron accumulation in thalassemia [7,8,9,10,11]. Females show greater resistance to heat and oxidative stress-induced cell death, likely because of the beneficial effects of estrogens, lower mitochondrial oxidative stress, and higher expression of genes involved in stress response [8,11]. Moreover, antioxidant enzymes, like superoxide dismutase (SOD), and reducing molecules, such as Glutathione (GSH), significantly increase in their expression and functions after exercise in females while not in males [12]. These findings have also been confirmed in mouse models showing that glutathione peroxidase and glutamate-cysteine ligase activities are higher in the kidneys and brains of female mice than in males, as well as SOD activity in female brains and lungs [13].

Crenotherapy with sulfureous mineral water exerts beneficial effects in several diseases and also improves cellular redox state in both animal models and humans [2,14,15,16,17,18,19,20]. Moreover, the sulfureous hydropinotherapy cycle significantly reduces circulating levels of reactive oxygen metabolites (ROMs) in patients with gastrointestinal disorders, likely protecting them from oxidative stress [21]. Hydropinotherapy relies on drinking mineral water in the morning by sipping during the fasting state to overcome possible interferences with digestive processes. Mineral water at room temperature or slightly heated should be assumed to avoid nausea or vomiting [21]. The type and the amount of mineral water to assume varies depending on the patient’s disease and age. The hydropinic cycle lasts for 12 days, always paused using a rest day in the middle of the therapeutic cycle. Conversely, mud-bath therapy uses mud applied on specific skin areas as 3–10 cm thick layers at a temperature of 41–50 °C and for 15–20 min, performed in the morning, in fasting conditions, and in specific rooms. During treatment, patients are covered with sheets, cellophane, and blankets to reduce rapid heat dispersion, and then mud is removed using a cleansing bath or a shower (at 37–38 °C) with tap or mineral water. At this stage, patients undergo a “reaction” step, as already described for balneotherapy. The therapeutic cycle consists of 12-day mud-bath applications with a rest day after 5–6 days of treatment [21,22,23].

Based on these considerations, in this retrospective observational study, we evaluated possible gender-related differences in the impact of sulfureous crenotherapy on plasma levels of ROMs and symptomatology in patients with rheumatic diseases and Vulgar psoriasis by stratifying for the first time by sex a real-life population treated with crenotherapy. We showed that sulfureous crenotherapy caused a more pronounced reduction of oxidant molecules in females with OA and psoriasis.

## 2. Results

### 2.1. Clinical Characteristics at Baseline

The study population (N = 78) was stratified by sex and included 40% of males (N = 31) with a mean age of 59 ± 9.5 years (range, 26–71 years) and a body mass index (BMI) of 27 ± 8.0, and 60% of females (N = 47) with a mean age of 57 ± 12.8 years (range, 28–81 years) and a BMI of 26 ± 5.1 (Table 1). No differences were found in age, BMI, and ROM levels at baseline between sexes, while OA was more frequent in females and psoriasis in males (*p* = 0.04). At baseline, 29% of females (N = 14) and males (N = 9) showed ROMs within normal values (mean ± SD, 267 ± 22 Carr.U. vs. 271 ± 29 Carr.U., females vs. males; *p* = 0.736). Conversely, the majority of subjects (71% in both sexes) displayed increased circulating ROM levels at baseline, and females tended to have higher ROMs compared to males (mean ± SD, 375 ± 53 Carr.U. vs. 348 ± 38 Carr.U., females vs. males; *p* = 0.059).

### 2.2. Plasma ROM Concentration

**Figure 1:**
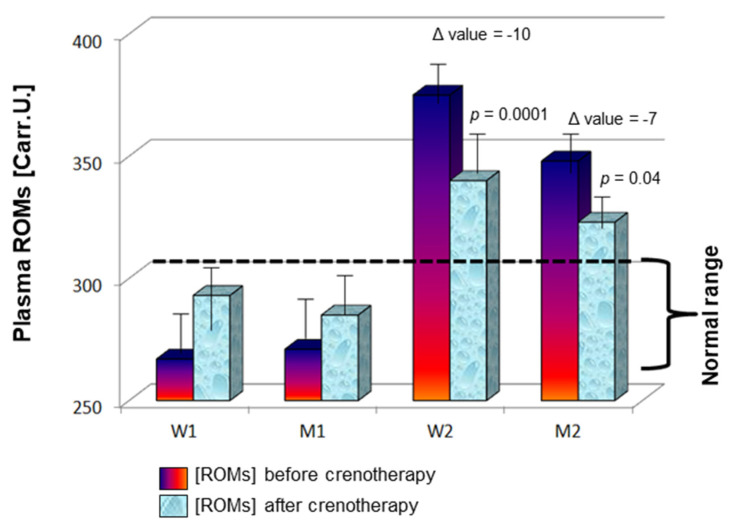
Mean ROM plasma levels ± SD at baseline and after 2 weeks of treatment in females and males with baseline normal ROM concentration (W1 and M1) or increased levels (W2 and M2). Delta *p* value was calculated as the difference between levels after 2 weeks of treatment and levels at baseline.

Next, ROM levels were monitored after the crenotherapy cycle and compared to baseline. No differences were observed after therapy for females and males with normal values at baseline (W1 and M1 group, respectively; Figure 1), as ROM levels were 293 ± 54 Carr.U. in females (vs. 267 ± 22 Carr.U. at baseline; *p* = 0.19), and 285 ± 38 Carr.U. in males (vs. 271 ± 29 Carr.U. at baseline; *p* = 0.16). Conversely, patients with high ROMs at baseline displayed a significant reduction after crenotherapy in both sexes (W2 and M2 group, respectively), more marked in females with a 10-fold reduction (mean ± SD, 375 ± 53 Carr.U. vs. 340 ± 65 Carr.U., before vs. after therapy; *p* = 0.0001) compared to males with a 7-fold decrease (mean ± SD, 348 ± 38 Carr.U. vs. 323 ± 60 Carr.U., before vs. after therapy; *p* = 0.04) (Figure 1).

### 2.3. Clinical Symptoms

After crenotherapy, clinical symptoms were significantly reduced compared to baseline in both sexes (*p* < 0.05) (Table 2), including only those subjects with high ROM levels at baseline (Table 3). Conversely, no differences were found in patients with normal ROM levels at baseline after therapy. In particular, in the total cohort, females at baseline complained more pain than males (VAS-score, mean ± SD, 2.8 ± 1 vs. 1.8 ± 1.1; *p* = 0.004), as well as morning stiffness (VAS-score, mean ± SD, 2.6 ± 1.4 vs. 1.0 ± 0.8; *p* = 0.008), while itch symptom was similar in both groups (VAS-score, mean ± SD, 1.8 ± 1.5 vs. 1.9 ± 1.8; females vs. males; *p* = 0.876). After crenotherapy, pain, morning stiffness, and itch improved in both sexes. VAS-scores for morning stiffness were lower in males (VAS-score, mean ± SD, 1.7 ± 1.4 vs. 0.5 ± 0.8; females vs. males; *p* = 0.042), while for itch in females (VAS-score, mean ± SD, 0.2 ± 0.4 vs. 1.0 ± 1.2; females vs. males; *p* = 0.030) (Table 2).

Similarly, in subjects with higher ROM levels at baseline, females complained of more pain (VAS-score, mean ± SD, 3.1 ± 0.8 vs. 2.0 ± 1.4; females vs. males; *p* = 0.020) and morning stiffness than males (VAS-score, mean ± SD, 3.0 ± 1.1 vs. 1.0 ± 0.8; females vs. males; *p* = 0.0001), while itch symptom was similar in both groups (*p* = 0.799). After crenotherapy, pain, morning stiffness, and itch improved in both sexes (Table 3). In particular, females showed a greater reduction in itching compared to males (−95% vs. −50%), with VAS-scores slightly lower in females than males (VAS-score, mean ± SD, 0.1 ± 0.4 vs. 1.0 ± 1.4; females vs. males; *p* = 0.077), while men had a higher amelioration in pain and morning stiffness compared to females (−45% vs. −32%, and −50% vs. −37%, respectively), with lower VAS-scores (VAS-score for pain, mean ± SD, 2.1 ± 1.2 vs. 1.1 ± 1.0; females vs. males; *p* = 0.063; and VAS-score for morning stiffness, mean ± SD, 1.9 ± 1.4 vs. 0.5 ± 0.76; females vs. males; *p* = 0.021).

Considering the entire duration of treatment, three males (5%) complained of belching (on the third day) and fatigue (during the last two days), while 10% of females experienced pain exacerbation (on the third day) and fatigue (on the last two days). All enrolled subjects completed the treatment.

## 3. Discussion

Crenotherapy or spa *salus per aquam* has therapeutic effects on various disorders, especially in those triggered by oxidative stress, and in some cases, they represent an alternative approach to standard pharmacological treatments [14,15,16,17,18,19,20,21,22]. Based on their ionic compositions, mineral waters can be classified as sulfureous, sulfate, bicarbonate, sodium chloride, ferrous arsenic waters, and others [23]. Sulfureous mineral waters containing combined sulfur (e.g., hydrogen sulfide) are used in mud-bath therapy for the treatment of arthro-rheumatic and musculoskeletal disorders [18,24,25,26]. Mud-bath therapy uses mud, a mixture of solid (organic and inorganic) and liquid (mineral water) components applied on specific skin areas, while hydropinotherapy with sulfureous mineral water relies on drinking mineral water for therapeutic aims [27,28]. Both treatments can reduce oxidative stress, as described in human and animal models [2,14,15,16,17,19,20]. Therefore, some studies also suggest combining both strategies to ensure long-lasting chondroprotective effects by persistently reducing oxidative stress, inflammation, and degradative stimuli [28].

In this retrospective observational real-life study, ROM plasma level changes were investigated after 2 weeks of sulfureous crenotherapy in a cohort of patients with OA or psoriasis stratified by sex. After crenotherapy, ROM plasma levels were significantly reduced in those subjects with increased ROMs at baseline, more marked in females than in males. This finding could be related to the antioxidant and anti-inflammatory effects of sulfureous mineral water used in crenotherapy (mud-bath therapy and hydropinotherapy). For example, sulfureous hydropinotherapy can provide the body’s supply of several minerals, like H_2_S, that have a cell signaling function and plays important roles in combating ROS and other free radicals [21,29,30]; magnesium, that helps to enhance efficiency of endogenous antioxidant systems [21,31]; and bicarbonate and calcium ions, that contribute to improvements of metabolism and motor-secretory activity of the gastrointestinal system [32,33]. In our cohort, ROM plasma levels in patients with arthro-rheumatic diseases and psoriasis Vulgaris plaque at baseline were similar to those reported in previous studies, confirming the consistency of this finding [15,17,21,34]. Therefore, the reduction of oxidant species in our cohort could be associated with the benefits of crenotherapy in improving clinical outcomes, such as reduced pain during daily activities, morning stiffness, and itch. In fact, mud-bath therapy has pain relieving, muscle relaxant, and decontracting actions and could ameliorate the quality of sleep, consequently rebalancing numerous metabolic functions [35,36,37]. In our study, we showed that improvements in clinical outcomes co-occurred at the end of the crenotherapy cycle in both sexes with the reduction of ROM plasma levels, especially in those subjects with higher oxidative stress at baseline. In particular, in this group of patients, females showed a greater reduction in itching compared to males (−95% vs. −50%), men had a higher amelioration in pain and morning stiffness compared to females (−45% vs. −32%, and −50% vs. −37%, respectively).

This study has some limitations: (i) the small sample size of each group and the absence of a control group, even though a reference ROM level range is already well-established; (ii) hydropinotherapy and mud-bath therapy were prescribed by different physicians, leading to treatment heterogeneity for similar conditions in our cohort; (iii) hormone levels or related signaling pathways were not investigated; and (iv) antioxidant status of subjects was not assessed to confirm the utility of sulfureous crenotherapy because of a small sample volume was available for each subject.

## 4. Materials and Methods

### 4.1. Patients and Study Design

A total of 78 Caucasian patients (mean age ± SD, 58 ± 11.6 years old) who received a sulfureous crenotherapeutic cycle at the Telese Spa (Benevento, Italy) were enrolled in this study after obtaining informed consent according to the Declaration of Helsinki and its amendments, and protocols approved by local Ethics Committee “Campania Sud” (no. 7 r.p.s.o./2020). Patients had a diagnosis of degenerative joint diseases (N = 51, including generalized OA, knee OA, and lumbar spine OA) or Vulgar psoriasis (N = 27) according to international guidelines (Table 4). Inclusion criteria for enrollment in this study were: age ≥18 years; history of rheumatic and dermatological chronic diseases; indications for crenotherapy; signed informed consent. Exclusion criteria were the presence of factors that could cause the increase in ROMs, such as acute clinical conditions, chronic diseases in the active phase, cancer, and voluptuous habits such as tobacco smoking.

### 4.2. ROM Measurement

Plasma ROM concentrations were measured at baseline and after 2 weeks of treatment using a d-ROMs test (Diacron International—Grosseto, Italy), a spectrophotometric assay for ROM level assessment, mainly hydroperoxides (ROOH) [3,33]. Normal plasma ROM levels were expressed as *Carratelli Units* (1 Carr.U. = 0.08 mg/L of H_2_O_2_) and ranged between 250–300 Carr.U. [33]. Pain and morning stiffness for chronic arthro-rheumatic diseases and itch for psoriasis were analyzed using the Visual Analogue Scale (VAS) with a score ranging from 0 (no symptom) to 10 (unbearable symptoms), as per international guidelines.

### 4.3. Statistical Analysis

Data were analyzed using the STATA 16 statistics package. Continuous variables, expressed as mean ± standard deviation (SD), were analyzed with t Student’s tests for paired and unpaired normally distributed data, while categorical variables using χ^2^ test. A *p* value < 0.05 was considered statistically significant.

## 5. Conclusions

In conclusion, spa therapy with mineral waters represents an alternative approach in musculoskeletal, skin, gastroenteric, and respiratory disorders because spa mineral waters influence several biological pathways via chemical stimuli, similar to common drugs. Therefore, sulfureous crenotherapy could be an effective alternative therapeutic strategy in OA and psoriasis treatment for both sexes, likely because of its antioxidant effects and in supplying the body with minerals important in muscle and bone homeostasis. Moreover, we showed for the first time that these crenotherapy-related effects could be more pronounced in females compared to males, probably because oxidative stress is also influenced by gender-related factors, like hormonal status. However, larger studies are needed to establish the type and extent of these beneficial effects from a gender-oriented perspective.

## Figures and Tables

**Table 1:** Baseline characteristics of the study population stratified by sex.

Characteristics	Females N = 47	Males N = 31	*p* Value
Age, years Mean ± SD Median (range)	57 ± 12.8 56 (8–81)	59 ± 9.5 61 (26–71)	0.459
BMI, Kg/m^2^ Mean ± SD (range)	26 ± 5.1 (21–41)	27 ± 8.0 (22–39)	0.502
ROMs at baseline, Carr.U. Mean ± SD (range)	348 ± 66.5 (237–495)	326 ± 50.0 (206–414)	0.125
Osteoarthritis (%)	74	52	0.04
Vulgar psoriasis (%)	26	48	0.04

Abbreviations. BMI, body mass index; ROMs, reactive oxygen metabolites.

**Table 2:** Mean values ± SD of evaluated clinical outcomes measured at baseline and after 2 weeks of crenotherapy in a total cohort of females and males.

Group		Females	Males	*p* Value
Pain (VAS-score)	before	2.8 ± 1.0	1.8 ± 1.1	0.004
	after	1.7 ± 1.2	1.0 ± 0.97	0.058
	*p* value	0.001	0.0003	
Morning Stiffness (VAS-score)	before	2.6 ± 1.4	1.0 ± 0.8	0.008
	after	1.7 ± 1.4	0.5 ± 0.8	0.042
	*p* value	0.002	0.04	
Itch (VAS-score)	before	1.8 ± 1.5	1.9 ± 1.8	0.876
	after	0.2 ± 0.4	1.0 ± 1.2	0.030
	*p* value	0.003	0.001	

**Table 3:** Mean values ± SD of evaluated clinical outcomes measured at baseline and after 2 weeks of crenotherapy in females (W2) and males (M2) with high ROM levels at baseline.

Group		W2	M2	*p* Value
Pain (VAS-score)	before	3.1 ± 0.8	2.0 ± 1.4	0.020
	after	2.1 ± 1.2	1.1 ± 1.0	0.063
	*p* value	0.0004	0.011	
Morning Stiffness (VAS-score)	before	3.0 ± 1.1	1.0 ± 0.8	0.0001
	after	1.9 ± 1.4	0.5 ± 0.76	0.021
	*p* value	0.003	0.04	
Itch (VAS-score)	before	2.2 ± 1.5	2.0 ± 1.95	0.799
	after	0.1 ± 0.4	1.0 ± 1.4	0.077
	*p* value	0.008	0.002	

Abbreviations. W2, females with increased reactive oxygen metabolites at baseline; M2, males with increased reactive oxygen metabolites at baseline.

**Table 4:** Characteristics of patients at baseline.

Characteristics	Population N = 78
Mean age ± SD, years	58 ± 11.6
Median age (range)	59 (28–81)
Gender, N (%)	
Males	31 (40)
Females	47 (60)
ROMs at baseline, Carr.U.	
Mean ± SD (range)	348 ± 66.5 (237–495)
Osteoarthritis, N (%)	51 (65)
Vulgar psoriasis, N (%)	27 (35)

Abbreviations. ROMs, reactive oxygen metabolites.

## Data Availability

Data are contained within the article.
